# Smart Polymers for Soft Materials: From Solution Processing to Organic Solids

**DOI:** 10.3390/polym15153229

**Published:** 2023-07-29

**Authors:** Debashish Mukherji, Kurt Kremer

**Affiliations:** 1 Quantum Matter Institute, University of British Columbia, Vancouver, BC V6T 1Z4, Canada; 2 Max Planck Institute for Polymer Research, Ackermannweg 10, 55128 Mainz, Germany; k.kremer@mpip-mainz.mpg.de

**Keywords:** polymer solution, smart polymers, coil–globule transition, solvent mixtures, organic solids, thermal conductivity

## Abstract

Polymeric materials are ubiquitous in our everyday life, where they find a broad range of uses—spanning across common household items to advanced materials for modern technologies. In the context of the latter, so called “smart polymers” have received a lot of attention. These systems are soluble in water below their lower critical solution temperature $T_{\ell }$ and often exhibit counterintuitive solvation behavior in mixed solvents. A polymer is known as smart-responsive when a slight change in external stimuli can significantly change its structure, functionm and stability. The interplay of different interactions, especially hydrogen bonds, can also be used for the design of lightweight high-performance organic solids with tunable properties. Here, a general scheme for establishing a structure–property relationship is a challenge using the conventional simulation techniques and also in standard experiments. From the theoretical side, a broad range of all-atom, multiscale, generic, and analytical techniques have been developed linking monomer level interaction details with macroscopic material properties. In this review, we briefly summarize the recent developments in the field of smart polymers, together with complementary experiments. For this purpose, we will specifically discuss the following: (1) the solution processing of responsive polymers and (2) their use in organic solids, with a goal to provide a microscopic understanding that may be used as a guiding tool for future experiments and/or simulations regarding designing advanced functional materials.

## 1. Overview

Just over a century ago, Hermann Staudinger, in a pioneering work, proposed how small molecules can join hands to form covalently connected long macromolecules, and this led to the foundation of modern synthetic polymer science [1]. Ever since this early discovery, the field of polymer science has come a long way, where various complex structures have been proposed for their use in designing high-performance functional materials for various desired applications [2,3,4,5,6,7,8,9,10,11]. Polymers are of particular interest, because they provide the basic building blocks for many modern materials of our daily life. This is particularly because polymers are a class of soft matter, where their relevant energy scale is of the order of $k_{B}T$ (at room temperature) [12,13,14], with $k_{B}$ being the Boltzmann constant, and, thus, they are dictated by large conformational and compositional fluctuations. This makes the processes of entropy as important as those of energy, and the microscopic understanding of this entropy–energy balance is at the heart of all soft materials design.

**Figure 1:**
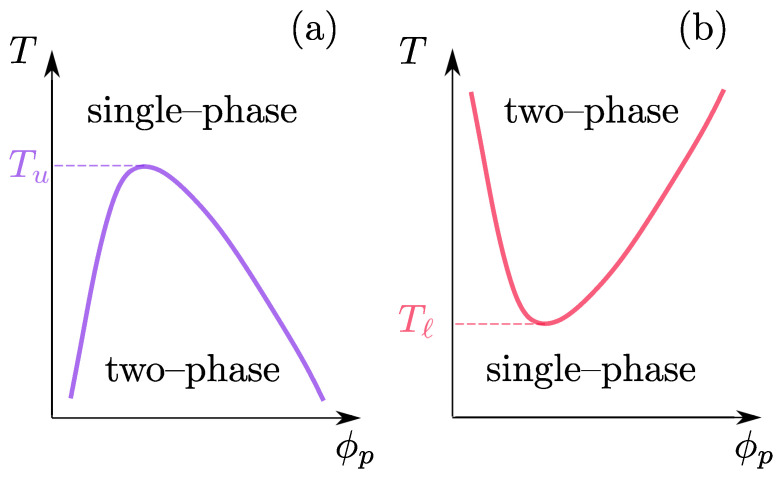
A simple schematic representation of the phase behavior of polymer solutions. Parts (**a**,**b**) show the upper critical (UCST) and the lower critical (LCST) solution temperature behaviors, respectively. Here, $\phi_{p}$ is the polymer volume fraction, $T_{u}$ is the UCST, and $T_{\ell }$ is the LCST.

Traditionally, most commonly used polymers have included those that are dominated by van der Waals (vdW) interactions; examples include, but are not limited to, polystyrene (PS), polyethylene (PE), polypropylene (PP), poly(N-acryloyl piperidine) (PAP), and poly(methyl methcrylate) (PMMA). Recent interests have been diverted towards biocompatible and thermoresponsive polymer architectures, which are often also referred to as “smart” polymers [15,16,17,18,19]. Some of the common examples of smart polymers include poly(acrylic acid) (PAA), polyacrylamide (PAM), poly(N–isopropyl acrylamide) (PNIPAM), and poly(vinyl alcohol) (PVA). Because of the hydrogen bond (H–bond) nature of the inter- and intramolecular interactions, the strength of which is between 4–8 $k_{B}T$ [20], these systems are soluble in water when the temperature *T* is lower than the “so called” lower critical solution temperature (LCST) $T_{\ell }$. For $T>T_{\ell }$, a chain undergoes a coil-to-globule transition [5,18,21,22,23,24]. This is because the increase in *T* breaks a fraction of polymer–water H–bonds, which thus also destroys the water caging around a polymer, which is responsible for keeping a chain expanded in water. In this process, the expelled water molecules, from within the first solvation shell of a polymer chain, gain translational entropy that is larger than the conformational entropy loss upon collapse, thereby making the LCST an entropy-driven process [12,13,14]. In the microgel community, $T_{\ell }$ is commonly known as the volume phase transition temperature $T_{\mathrm{VPTT}}$ [25,26,27]. On the contrary, when a chain expands upon an increase in *T*, it is known as the upper critical solution temperature $T_{u}$ and is driven by energy. In Figure 1, we show the generic phase diagrams of polymer solutions with a UCST (part a) and an LCST (part b). Note thatm unless stated otherwise, we will primarily focus on LCST polymers in this review.

Starting from a good solvent condition in an LCST collapse (i.e., for $T<T_{\ell }$), increasing the effective attraction between monomers causes the polymer to eventually collapse into a globule in the case of isolated chains (or phase separation in solutions). This globular state is dictated by balancing the attractive second virial contributions −|V| and the three-body repulsion [12,13,14], where V is the monomer-excluded volume. Here, a conventional Θ collapse of a polymer is characterized as a second-order phase transition, where the Θ point (or the critical point) is dictated by the large diverging fluctuations. However, there are also many cases where hysteresis is observed near $T_{\ell }$ [21,28,29], which is a typical indication of a first-order transition [30,31]. In the case of LCST polymers, first-order [21,28,29], as well as second-order [22] behavior, can be observed.

**Figure 2:**
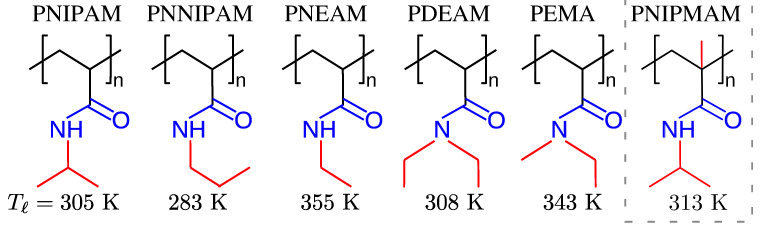
Schematic representation of different homopolymer monomer structures with slight changes in their hydrophobic side groups (represented by red). One special case of poly(N-isopropyl methacrylamide) (PNIPMAM) is also shown (within the dashed gray box) that has almost the same chemical structure as poly(N-isopropyl acrylamide) (PNIPAM), with an additional a methyl group attached to the backbone; see the text for more details. The corresponding values of the lower critical solution temperature $T_{\ell }$ are also highlighted.

The most commonly known examples of LCST polymers are poly(ethylene oxide) (PEO) and/or poly(N–isopropyl acrylamide) (PNIPAM) in aqueous solutions. While PEO has a $T_{\ell }$ that is almost close to the boiling temperature of water [32,33,34], PNIPAM has $T_{\ell }≃305$ K [5,21,23], i.e., a temperature just below the human body temperature. This makes PNIPAM a very interesting and a widely studied polymer. It is also noteworthy that the actual value of $T_{\ell }$ of a homopolymer can be significantly tuned through a slight change in the monomeric chemical structures [24]; see Figure 2. Typically, increasing the size of the hydrophobic side group reduces the $T_{\ell }$. However, one special system is poly(N-isopropyl methacrylamide) (PNIPMAM), where an additional methyl group attached to the backbone of the PNIPAM increases the $T_{\ell }$ to about 313 K for the PNIPMAM in comparison to the $T_{\ell }≃305$ K for the PNIPAM [35]. At first sight, this is, at least, counterintuitive and still not readily understood. However, one possible scenario might be that the local chain conformation of the PNIPMAM becomes affected by this methyl side group and effectively reduces the hydrophobic surface area of the chains.

Tuning the $T_{\ell }$ can not only be obtained in the homopolymer structures, but instead through a possibly better and more versatile protocol, which is to use the copolymer sequences that might provide a greater control on the tunability of the $T_{\ell }$ [16,22,24,36,37,38,39,40]. A common technique to increase the $T_{\ell }$ is the introduction of more hydrophilic monomers along a polymer backbone [22,23,41,42], while the $T_{\ell }$ can be reduced by introducing hydrophobic monomers [22,42]. Here, however, in most cases the interaction of the solvent molecules with one monomer type becomes greatly affected by the solvation structures of the neighboring monomers. This induces a strong crosscorrelation between the different monomer species and, thus, the shift in the $T_{\ell }$ remains rather unpredictable (nonlinear) [23,41,42].

**Figure 3:**
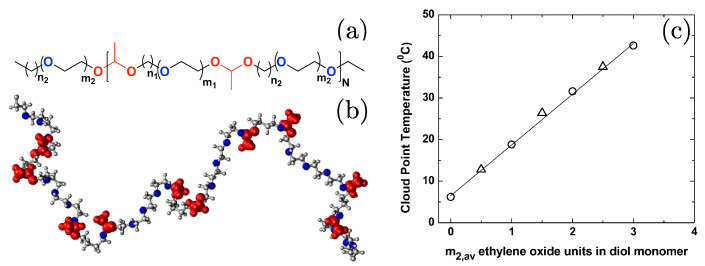
Parts (**a**,**b**) show the schematic of an experimentally synthesized polyacetal chain [22] and the corresponding simulation snapshot at 290 K [43], respectively. The number of hydrophobic $n_{i}$ and hydrophilic $m_{i}$ units were tuned to obtain the desired cloud point temperature $T_{\mathrm{cloud}}$. The acetal linkers are represented in red. Part (**c**) shows the change in $T_{\mathrm{cloud}}$ as a function of $m_{2}$. Parts (**a**,**b**) have been reproduced with permission from the American Institute of Physics [43], and part (**c**) has been reproduced with permission from the American Chemical Society [22].

A recent experimental study proposed a set of copolymers consisting of ethylene oxide and methylene units, where a linear variation in the cloud point temperature $T_{\mathrm{cloud}}$ was observed with the change in the relative variation in monomer concentrations [22,43]. The chemical structure and the variation in the $T_{\mathrm{cloud}}$ are shown in Figure 3. The predictability of the $T_{\mathrm{cloud}}$ (or $T_{\ell }$) comes from the linear variation in the $T_{\mathrm{cloud}}$; see Figure 3c. For example, if one requires a particular value of $T_{\mathrm{cloud}}$, data such as the ones from Figure 3c can be used to estimate what relative fraction of ethylene oxide and methylene is needed. The added advantage of this system is that the monomers are connected by the acetal links and, thus, are commonly referred to as polyacetals; see Figure 3a [22]. While polyacetals are LCST thermoresponsive, they are also biodegradable because of their acetal linkage.

**Figure 4:**
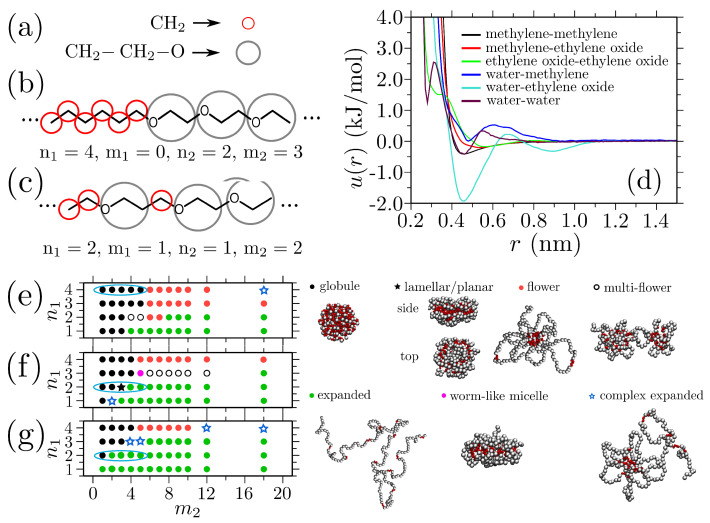
Parts (**a**–**c**) show the mapping scheme and the corresponding coarse-grained (CG) representations of two polyacetal chains with different amphiphilic sequences. Part (**d**) shows the sequence-transferable CG potentials between different pairs. Finally, parts (**e**–**g**) show the phase diagrams of 144 chains with different sequences and their corresponding configurations. This figure has been reproduced with permission from the American Institute of Physics [43].

The above experiments are very important to provide a guiding path, but the range of the relative monomeric compositions investigated in Ref. [22] remains rather limited because of the synthesis-related limitations. Therefore, simulations (especially the multiscale approaches) may provide a better platform to explore a large range of copolymer configurations with minimalistic computational costs. In this context, a segment-based (i.e., monomer level) coarse-grained (CG) model was developed to investigate the conformational behavior of a broad range of polyacetals [43]; see Figure 4. This structure-based CG model was derived using a combination of the iterative Boltzmann inversion [44,45] and the coordination IBI [46]. Furthermore, this approach makes use of the linear variation of the $T_{\mathrm{cloud}}$ with the sequence length (shown in Figure 3c), which highlight that there is no crosscorrelation between the solvation structures of the two monomer species constituting a polyacetal. Therefore, intermolecular CG potentials were derived at the monomer level (shown in Figure 4d), and their derivations were sufficient to explore the conformational behavior of a large set of polyacetal sequences; see Figure 4e–g. Additionally, other than the consistency with the existing experimental data (highlighted by the cyan oblique circles in Figure 4e–g) [22], this model also found very interesting copolymer structures based on their relative sequences; see the simulation snapshots in Figure 4. We also note in passing that, for the other sequences, such as the nonblock random sequences, the above mentioned CG protocol might need some more fine-tuning.

The discussions presented above deal with a reasonably broad range of standard polymer architectures. In addition to these, another set of architectures includes the peptide-based systems that can also mimic the LCST-type phase behavior [15,47]. A common example of peptide-based systems are the elastin-like polypeptides (ELP), which are characterized by the standard sequence (**VPGXG**)–. Here, V, P, and G are valine, proline, and glycine, respectively. The residue **X** can be anything but proline. The $T_{\ell }$ of an ELP sequence can be easily tuned by taking an appropriate **X** [48,49,50]. Another advantage of ELPs is that the $T_{\ell }$ can be further tuned by proline isomerization [51,52], where *cis* and *trans* configurations can significantly alter the water caging around the ELP, which consequently changes its $T_{\ell }$.

While the conformational tuning by changing *T* yields a flexible route for the solution processing of “smart” polymers and ELPs, it often requires an unusual variation in *T* and, thus, poses a challenge for the practical use of these polymers. Therefore, a possible alternative might be to use cosolvents as an external stimulus at a fixed *T*. In this context, it has been well known that the structure, function, and stability of a polymer in water become severely affected by the presence of small molecules [53,54,55,56,57,58,59,60,61,62,63,64,65,66,67,68,69,70] and/or ions [17,71,72] within the first solvation shell.

The importance of polymer processing in mixed solvents was already highlighted over half a century ago [73,74,75]. Here, polymer solvation in binary mixtures often appears paradoxical, where a delicate balance of the microscopic interactions between different solution components dictates the macroscopic conformational behavior. Two such phenomena are *co-nonsolvency* and *cosolvency*. Therefore, in the following, we will now focus on polymer solvation in binary mixtures by reviewing two symmetric effects and their thermodynamic origins within the context of multiscale simulations.

## 2. Co-Nonsolvency

**Figure 5:**
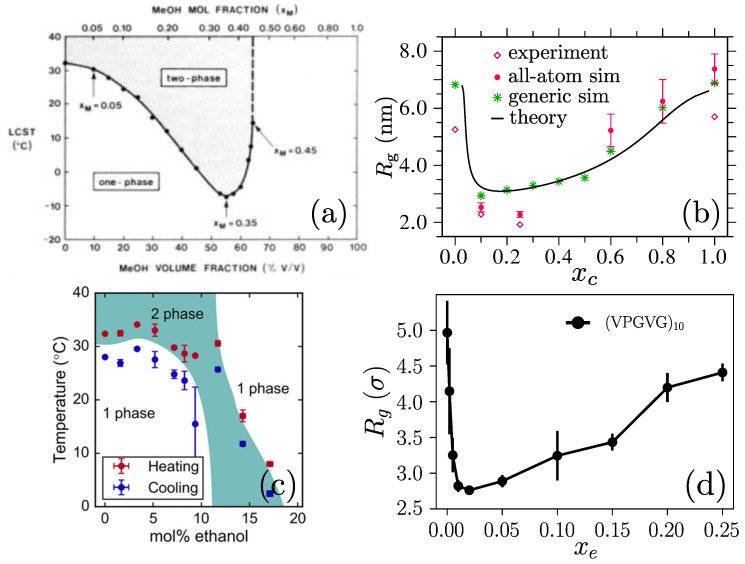
The phase diagrams for a poly(N–isopropyl acrylamide) (PNIPAM) [54]: (part (**a**)) and an elastin-like polypeptide (ELP) [77] (part (**c**)) in aqueous alcohol mixtures. The corresponding single-chain gyration radii $R_{g}$ for PNIPAM (part (**b**)) and ELP (part (**d**)) are also shown. While $R_{g}$ calculated from the different techniques are shown for PNIPAM [76] in panel (**b**), ELP data were calculated using generic simulations [78]. Figures in parts (**a**,**c**,**d**) have been reproduced with permission from the American Chemical Society [54,77,78] and part (**b**) has been reproduced with permission from the Royal Society of Chemistry [76].

Co-nonsolvency is a generic name given to the phenomenon of polymer collapse in the mixtures of two miscible good solvents. This phenomenon was initially reported for PS solvation in the mixtures of cyclohexane and N,N-dimethylformamide (DMF) [75], where the name co-nonsolvency was initially coined. Even though this phenomenon was discovered for a UCST polymer, it gained popularity in the context of PNIPAM solvation in aqueous alcohol mixtures [53,54]. PNIPAM is a “well known” thermoresponsive polymer that shows LCST phase behavior in pure water. In aqueous alcohol mixtures, the $T_{\ell }$ of a PNIPAM system first decreases with the increasing alcohol (or cosolvent) mole fraction $x_{c}$ and again increases sharply around $x_{c}≃0.40$; see Figure 5a. More specifically, when a small amount of alcohol is added in water at a fixed *T* (for example at T=300 K), a PNIPAM chain collapses and then again opens up when $x_{c}\geq 0.40$ [55,76]; see Figure 5b.

It is not only that the PS and PNIPAM show co-nonsolvency-like phase behavior. A rather broad range of polymers shows similar solvation behavior in their respective binary solutions [56,57,79,80,81,82,83]. This list also includes standard ELPs; see Figure 5c,d for the phase behavior obtained in experiments [77] and the corresponding conformational transition from a generic simulation model [78]. One special case, however, is PDEAM in aqueous–alcohol mixtures; see the chemical structure in Figure 2. It was shown that PDEAM does not show co-nonsolvency in aqueous methanol [84], but it collapses in aqueous ethanol [82]. We also wish to point out that the range of co-nonsolvency collapse is a *T*, molecular weight $M_{w}$, and cosolvent-dependent quantity [85,86,87].

If two solvents are good for a polymer and also remain fairly miscible, why should a polymer collapse within certain combinations of such mixed solvents? In recent times, there has been considerable interest to investigate the phenomenon of co-nonsolvency using a broad range of experimental, computational, and theoretical techniques. In particular, analytical and simulation efforts have been devoted to unveiling the microscopic origin of co-nonsolvency, where three main mechanisms have been proposed, namely, the Flory–Huggins mean field description [53], the cooperativity effect [58,85], and preferential binding [63,88], which we will now review in the context of their complementary experiments.

### 2.1. Flory–Huggins Mean Field Description

A standard mean field thermodynamic description of polymer conformation is the Flory–Huggins (FH) theory [12,13,14]. Here, when a polymer *p* with a chain length $N_{l}$ at a volume fraction $\phi_{p}$ is dissolved in a binary mixture of the solvent *s* and cosolvent *c*, the free energy $F_{\mathrm{FH}}$ is written as follows [12,13,14]:

(1) $$\begin{array}{cc} \frac{F_{\mathrm{FH}}}{\kappa_{B}T} & =\frac{\phi_{p}}{N_{l}}\ln\phi_{p}+x_{c}\left(1-\phi_{p}\right)\ln\left[x_{c}\left(1-\phi_{p}\right)\right]\\ \; & +\left(1-x_{c}\right)\left(1-\phi_{p}\right)\ln\left[\left(1-x_{c}\right)\left(1-\phi_{p}\right)\right]\\ \; & +\chi_{ps}\phi_{p}\left(1-x_{c}\right)\left(1-\phi_{p}\right)\\ \; & +\chi_{pc}\phi_{p}x_{c}\left(1-\phi_{p}\right)\\ \; & +\chi_{sc}x_{c}\left(1-x_{c}\right){\left(1-\phi_{p}\right)}^{2}. \end{array}$$

The first three and the last three terms represent the mixing entropy and the interaction between different solution components, respectively. $\chi_{ij}$ is the interaction parameter between the components *i* and *j*. The second-order expansion of Equation (1) yields the estimate of the polymer-excluded volume V (or the second virial):

(2) $$\begin{array}{c} V=1-2\left(1-x_{c}\right)\chi_{ps}-2x_{c}\chi_{pc}+2x_{c}\left(1-x_{c}\right)\chi_{sc}. \end{array}$$

Furthermore, $V=-2\pi \int \left[e^{-v\left(r\right)/k_{B}T}-1\right]r^{2}dr$ gives a direct indication of the solvent quality via the effective monomer–monomer interaction v(r). For example, in a good solvent condition, V>0, where the single-chain structure factor follows a scaling relation $S\left(q\right)∼q^{-5/3}$. Increasing the monomer–monomer attraction brings a polymer into the Θ condition, where V=0, and $S\left(q\right)∼q^{-2}$. A further increase in the monomer–monomer attractions the takes a system to the globular state, where V<0, and $S\left(q\right)∼q^{-4}$ [12,13,14].

**Figure 6:**
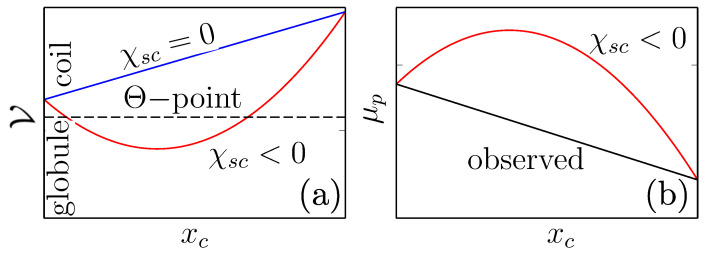
A schematic representation of the polymer-excluded volume V (part (**a**)) and chemical potential $\mu_{p}$ (part (**b**)) as a function of cosolvent mole fraction $x_{c}$. The curves are shown for different solvent–cosolvent interactions $\chi_{sc}$. The Θ point corresponds to V=0. This figure has been reproduced with permission from the American Institute of Physics [89].

The description in Equation (1) suggests that $\chi_{ij}<1/2$ for a good solvent polymer solution [12,13,14]. Furthermore, when two cosolvents are perfectly miscible (i.e., $\chi_{sc}=0$), the first two terms of Equation (2) yield a linear variation in V with $x_{c}$; see the blue line in Figure 6a. Only when $\chi_{sc}<0$ can co-nonsolvency can be observed (i.e., V<0); see the red line in Figure 6a.

Under the infinite dilution limit of a polymer (i.e., $\phi_{p}\to 0$), Equation (1) also gives an estimate of the polymer chemical potential $\mu_{p}$:

(3) $$\begin{array}{cc} {\bar{\mu }}_{p}\left(\phi_{p}\to 0\right) & =\frac{\partial F_{\mathrm{FH}}}{\partial \phi_{p}}{|}_{\phi_{p}\to 0}\\ \; & =\mathrm{const}-x_{c}\ln x_{c}-\left(1-x_{c}\right)\ln\left(1-x_{c}\right)\\ \; & +\left(1-x_{c}\right)\chi_{ps}+x_{c}\chi_{pc}-2x_{c}\left(1-x_{c}\right)\chi_{sc}. \end{array}$$

When $\chi_{sc}<0$, Equation (3) yields the expected variation shown by the red line in Figure 6b, i.e., it becomes energetically expensive to solvate a polymer within the intermediate range of $x_{c}$ where a polymer collapses. As we will show in Section 2.3, the observed trend in $\mu_{p}$, represented by the black line in Figure 6b, requires an unrealistic cost of driving the bulk binary mixture to phase separation by using $\chi_{sc}>>0$.

Note that when the solvent–cosolvent interaction becomes attractive (i.e., $\chi_{sc}<0$), it is quite obvious why a polymer falls out of the solution [53,65,90]. However, it is readily known that the common solvent mixtures where co-nonsolvency is observed, such as the water–alcohol mixtures, both (co)solvents are typically only fairly miscible, i.e., $\chi_{sc}≃0$. Indeed, the experimental [91,92] and the simulation [27,93] data for aqueous alcohol mixtures are in agreement with this argument. Therefore, it is not quite obvious how the standard FH-based mean field theory can lead to the phenomenon of co-nonsolvency. However, it has been discussed that, if the three-body interactions are considered, co-nonsolvency can be observed within the mean field description [94,95].

### 2.2. Cooperativity Effect

An alternative theoretical approach to describe the co-nonsolvency phenomenon is the cooperativity effect [58,85]. This approach was predominantly developed for PNIPAM and described its LCST response [96]. This approach considers the following phenomena: (i) the formation an H–bond between one water molecule and a monomer, which cooperatively facilitates the formation of the next H–bonds. (ii) The backbone segments that are not bound to the water aggregate into a collapsed region. This model was then extended to describe the co-nonsolvency phenomenon where both water molecules and alcohols were treated equally, therein estimating the interaction parameters accounting for different affinities and cooperativity effects. This approach used a set of parameters that could very well describe the experimental results of PNIPAM solvation in water–methanol mixtures [58] or some mixed solvents in general [66,85,97].

### 2.3. Preferential Interactions

**Figure 7:**
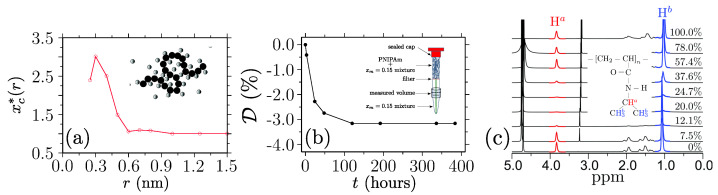
Part (**a**) shows the normalized methanol mole fraction $x_{c}^{*}=x_{c}\left(r\right)/x_{c}$ as a function of the radial distance *r* from a poly(N–isopropylacrylamide) (PNIPAM) backbone. In a nutshell, an excess occurs when $x_{c}^{*}>1$, and $x_{c}^{*}=1$ represents the bulk mixing ratio $x_{c}$. This dataset is shown for $x_{c}=0.1$. In the inset of part (**a**), a schematic of polymer (black circles) is shown in the presence of cosolvent molecules (gray circles). Part (**b**) shows the percentage of excess methanol molecules absorbed by the PNIPAM sample in the upper part of the nuclear magnetic resonance (NMR) measurement tube (see the inset). A depletion of methanol content in the lower compartment of the NMR tube gives a direct estimate of the methanol intake in the PNIPAM. The data is shown for $x_{c}=0.15$. Finally, part (**c**) presents the proton NMR spectra of the hydrogen atoms (highlighted in the inset) as a function of $x_{c}$. Reduced intensity is a measure of the dynamics of the PNIPAM side group. These figures have been reproduced with permission from the Royal Society of Chemistry [76].

Another approach to describe the phenomenon of co-nonsolvency uses the idea of preferential polymer–cosolvent binding with respect to the solvent–polymer interactions. As seen from the all-atom simulations in Figure 7a, there is an excess of cosolvents within the first solvation shell of a PNIPAM chain, i.e., when $x_{c}^{*}>1$ for r≤0.6 nm [98]. More specifically, when a small amount of cosolvent molecules is added in a polymer–solvent solution, they cluster around the monomers because of their preferential binding and promote the aggregation of monomers to minimize the binding free energy. In this process, cosolvent molecules form sticky contacts between monomers that are topologically far along a chain backbone that is facilitated by the formation of segmental loops; see the inset in Figure 7a [88].

Within a simple scaling argument, formation of a loop of segment length *n* can be characterized by the free energy difference ▵F(n) to form a loop from an extended conformation. Here, the partition function of an expanded chain of length $N_{\ell }$ and with an end-to-end distance $R_{\mathrm{ee}}$ can be defined as follows [14]:

(4) $$\begin{array}{c} Z\left(R_{\mathrm{ee}}\right)\propto Q^{N_{\ell }}N_{\ell }^{\gamma -1}, \end{array}$$

and for the case when $R_{\mathrm{ee}}\to 0$,

(5) $$\begin{array}{c} Z\left(R_{\mathrm{ee}}\to 0\right)\propto Q^{N_{\ell }}N_{\ell }^{\alpha -2}. \end{array}$$

The 1/Q value is the critical fugacity, and γ=1.15 and α=0.2 are the critical exponents [12,13,14]. Following these definitions, one can estimate $▵F\left(n\right)=mk_{B}T\ln\left(n\right)$ with m=1.95. This estimate suggests that the energy penalty to form a short segmental loop is of the order of a few $k_{B}T$ [88,89].

The preferential binding picture is supported by the proton NMR experiments [76]. For example, as measured by the depletion D of cosolvent molecules in the lower compartment of an NMR tube (see Figure 7b), the same amount of cosolvents were absorbed by the PNIPAM sample in the upper compartment of the NMR tube; see the inset of Figure 7b. Note that the sealed top cap prevented the evaporation of methanol molecules, as also shown by the plateau in Figure 7b for t>150 h.

The excess of cosolvent molecules populated the side groups of the PNIPAM. Something that speaks in this favor is that the side groups of the PNIPAM became more rigid within the range of $10\%\leq x_{c}\leq 40\%$, i.e., when a PNIPAM collapsed. Furthermore, the side groups organized in such a way that they were buried inside a collapsed PNIPAM, encapsulated by a certain amount of methanol molecules, and, thus, formed a fluffy PNIPAM globule [76]. Note that this was a different outcome from the standard picture of polymer collapse in poor solvents, which will be discussed in detail at a later stage in this review. The solvation of PNIPAM in aqueous urea mixtures also shows a similar preferential binding trend. In this context, it has been shown that a PNIPAM chain collapses in aqueous urea by forming H-bonded bridging urea molecules that bind to two monomers far along a chain backbone [17,67].

Note also that earlier studies have highlighted the importance of preferential binding to describe polymer solvation in binary mixtures [74,99,100]. A recent work also emphasized that the preferential binding may not be a prerequisite for co-nonsolvency [101], which may, however, require strong solvent–cosolvent interactions.

**Figure 8:**
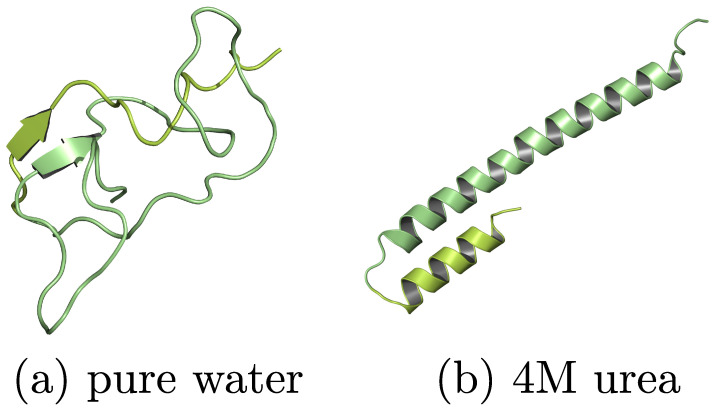
Simulation snapshots showing the secondary structure of a polyalanine in pure water (part (**a**)) and in 4 M aqueous urea mixture (part (**b**)). A clear indication of stable secondary structure is shown in 4 M aqueous urea mixtures. This figure has been reproduced with permission from the American Chemical Society [110].

The above discussions focused on describing general polymer conformations (i.e., going from a random coil to a collapsed globule) without specifically ordered regions. However, cosolvent effects are also most commonly associated with the ability of proteins and polypeptide sequences to form well-defined secondary structures [102,103,104,105,106,107,108,109]. In this context, urea is a common osmolyte that is known to denature native protein structures via preferential binding [103,104,105,106,108,109]. On the contrary, studies have also indicated that a certain set of polypeptides (such as polyalanine or alanine–rich sequences) may show the signatures of folding in aqueous urea mixtures [102,107,110]. It can be appreciated from the snapshots of Figure 8 that a polyalanine remains in a rather globular conformation in pure water (part a), and a well-defined secondary structure can be observed in 4 M aqueous urea mixtures (part b). Furthermore, simulation results have suggested that the preferential binding alone cannot account for this behavior; rather, a delicate balance between the preferential peptide–urea H–bond and the dipole–dipole interactions (DDIs) controls the conformational transition of polyalanines in aqueous urea mixtures [110]. This interpretation is of particular importance, because DDIs aew known to play a key role in protein solvation, as highlighted in a recent experimental study [111], yet they are poorly investigated in the existing literature.

#### 2.3.1. Solvation Thermodynamics: Kirkwood–Buff Theory of Solution

Polymer–cosolvent preferential binding can also result in an interesting thermodynamic trend, as estimated by the variation in the polymer chemical potential $\mu_{p}$ with $x_{c}$. A direct method to calculate $\mu_{p}$ is the fluctuation theory of Kirkwood and Buff (KB). The KB theory is an extremely powerful tool to calculate the solvation properties of complex multicomponent mixtures, and it has been extensively used in studying solvent mixtures [91,112,113,114], polymer solutions [63,115], ionic systems [116,117], and/or long-chain polymer blends [118,119].

In a nutshell, the KB theory connects the fluctuations in a grand canonical ensemble (i.e., a constant solution chemical potential μ, constant volume V, and constant T ensemble) to pair distribution functions $g_{ij}^{\mu VT}\left(r\right)$ via the “so called” Kirkwood–Buff integral (KBI) [91,112]:

(6) $$\begin{array}{cc} G_{ij} & =4\pi \int_{0}^{\infty }\left[g_{ij}^{\mu VT}\left(r\right)-1\right]r^{2}dr\\ \; & =V\left[\frac{\langle N_{i}N_{j}\rangle -\langle N_{i}\rangle \langle N_{j}\rangle }{\langle N_{i}\rangle \langle N_{j}\rangle }-\frac{\delta_{ij}}{\langle N_{j}\rangle }\right]. \end{array}$$

Here, $G_{ij}$ is known as the excess coordination between two different species *i* and *j* that are blended in at different $x_{j}$. In polymer physics, $V_{ij}=-G_{ij}/2$ gives an estimate of the monomer-excluded volume. The thermal averages are denoted by brackets $\langle \cdot \rangle$. $N_{i}$ is the number of particles of species *i*, and $\delta_{ij}$ is the Kronecker delta. To precisely calculate $G_{ij}$, the correlations have to be integrated to r→∞. However, within the midsized and closed boundary simulation setups [112], $G_{ij}$ is usually calculated from the *r* values that are slightly larger than the typical correlation lengths $r_{\mathrm{corr}}>1.5$ nm in the aqueous systems. We also note in passing that there are several more sophisticated simulation protocols that usually deal with semigrand canonical schemes [63,120] or by using fluctuations [114,121,122].

**Figure 9:**
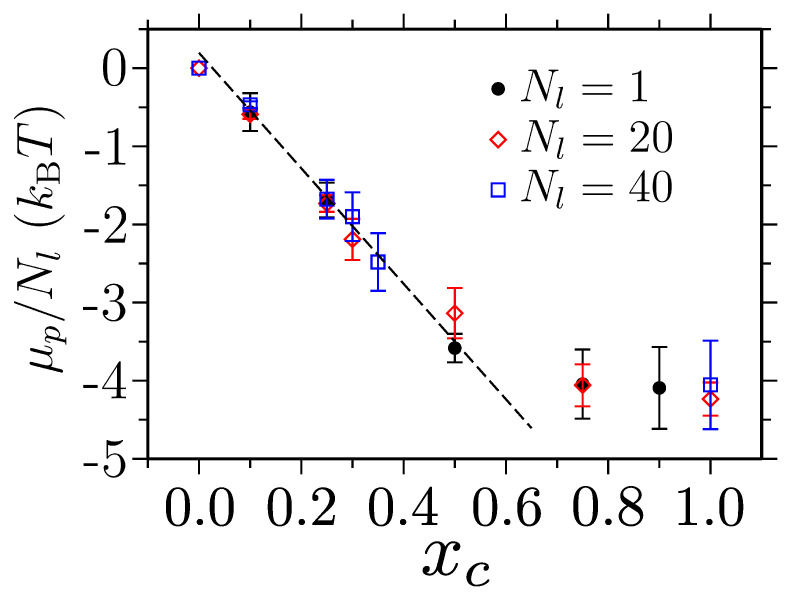
The shift in polymer chemical potential $\mu_{p}$ per monomer as a function of methanol mole fraction $x_{c}$. Data are shown for a poly(N–isopropyl acrylamide) (PNIPAM) in water–methanol mixtures with three different chain lengths $N_{l}$ at a temperature T=300 K. This figure has been reproduced with permission from the American Chemical Society [63].

Within the framework of KB theory, $\mu_{p}$ can be calculated using the following formula [63,91,123]:

(7) $$\lim_{\phi_{p}\to 0}\frac{\partial \mu_{p}}{\partial x_{c}}=\frac{k_{B}T\left(G_{ps}-G_{pc}\right)}{1-\rho_{c}\left(G_{sc}-G_{cc}\right)},$$

with $\rho_{c}$ being the number density of the cosolvents. Figure 9 shows the shift in $\mu_{p}$ as a function of $x_{c}$.

It can be appreciated that, upon adding cosolvent molecules (in this case, methanol) in aqueous–PNIPAM solutions, the solvent quality becomes better and better [63]. Furthermore, data have also revealed that, in this case, there was a contrast of about 4$k_{B}T$ between the solvent–polymer and the cosolvent–polymer interactions; thus, the cosolvent molecules were a significantly better good solvent for the polymer than the solvent molecules [63,88].

The behavior observed in Figure 9 is different from what would be expected from the mean field description of polymer solutions; see the red dataset in Figure 6b, i.e., when a polymer collapses, it should become energetically costlier to solvate. This stark qualitative difference suggests that the polymer conformation and its thermodynamic state are not connected in the conventional way, thus presenting a need for an analytical treatment that goes beyond the standard Flory–Huggins mean field description [53,89].

We also highlight that the shift in $\mu_{p}$ (observed in Figure 9) can be used to develop thermodynamically consistent generic models. This can be achieved by simply treating the interparticle interactions based purely on the Lennard–Jones potential and then tuning the relative parameters between different solution components that reproduce the reference all-atom $\mu_{p}$. This approach can reasonably capture the solvation behaviors of PNIPAMs [88] and ELPs [78] in aqueous–alcohol mixtures.

#### 2.3.2. Competitive Displacement of Solvents by Cosolvents

It has been demonstrated that co-nonsolvency has been observed for a broad range of chemical specific systems [53,54,56,57,77,80,81,82], which thus suggests that there may be a more generic microscopic picture of this complex solvation behavior. In this context, a simple generic picture was proposed that made use of the idea of preferential cosolvent–monomer interactions, as shown in Figure 7 and Figure 9. This treatment considers a polymer as a surface with N adsorbing sites, where both solvents and cosolvents compete to be adsorbed, as described within the Langmuir-like adsorption isotherm [124]. This is in contrast to the mean field picture of polymer solution, where the (co)solvents effects are treated as the average field, irrespective of their spatial distributions around a polymer.

**Figure 10:**
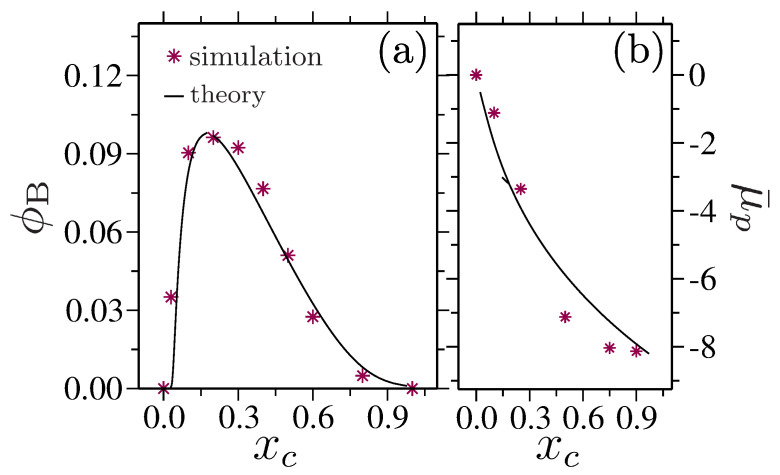
Part (**a**) shows the fraction of bridging cosolvents $\phi_{B}$ as a function of cosolvent mole fraction $x_{c}$. $\phi_{B}$ forms sticky contacts between two monomers topologically far along the chain contour. Part (**b**) shows the shift in polymer chemical potential ${\bar{\mu }}_{p}$ as a function of $x_{c}$. The lines are the results of obtained from the analytical model in Equations (9) and (10). Datasets have been taken from Refs. [88,89].

When a polymer collapses under the influence of a cosolvent, a collapsed globule contains a large fraction of cosolvent molecules that bind to more than one monomer to form a sticky bridging contact; see the schematic in the inset of Figure 7a. Note that a cosolvent molecule is considered to be confined by the local polymer environment when its residence time is longer than the time it takes for the polymer to move its own size. In this case, a polymer globule contains a fraction of bridging cosolvents $\phi_{B}$, a fraction of cosolvents that are adsorbed on a single site ϕ, and the remaining $1-\phi_{B}-\phi$ sites are occupied by the solvent molecules. As expected, the $\phi_{B}$ bridging cosolvents show a hump within the range of $x_{c}$ when a polymer collapses; see the simulation data in Figure 10a. Here, the individual bridging contacts consist of a few cosolvent molecules, and this is not only by one cosolvent [88].

Within the adsorption isotherm picture, the free energy per site Ψ can then be written as follows:

(8) $$\begin{array}{ccc} \frac{\Psi }{\kappa_{B}T} & = & \phi \ln\left(\phi \right)+2\phi_{B}\ln\left(2\phi_{B}\right)\\ & + & \left(1-\phi -2\phi_{B}\right)\ln\left(1-\phi -2\phi_{B}\right)\\ & - & E\phi -E_{B}\phi_{B}-\frac{\mu }{\kappa_{B}T}\left(\phi +\phi_{B}\right). \end{array}$$

The first three terms are the mixing entropic contributions, and the factor of two represents the simple picture that a collection of bridging cosolvents bind to two monomers. The next two terms are related to the adsorption energies of the bridging $E_{B}$ and single site E cosolvent molecules. $\mu =k_{B}T\ln\left(x_{c}\right)$ is the cosolvent chemical potential in the bulk solution at $x_{c}$. Furthermore, $E_{B}=2E-mk_{B}T\ln\left(n\right)$, i.e., $E_{B}$ is reduced with respect to 2E by the cost of forming a loop of the segmental length *n*, which was discussed earlier in this review. By minimally mixing Equation (8) one obtains the following:

(9) $$\begin{array}{ccc} 16{\phi_{B}}^{2}x_{c} & = & x_{c}^{a}\{{\left(\frac{x_{c}^{a}}{x_{c}^{b}}\right)}^{1/2}\phi_{B}^{m/2}\left(1-2\phi_{B}\right)\\ & \pm & \sqrt{\left(\frac{x_{c}^{a}}{x_{c}^{b}}\right)\phi_{B}^{m}{\left(1-2\phi_{B}\right)}^{2}-16{\phi_{B}}^{2}}{\}}^{2}, \end{array}$$

where $x_{c}^{a}=e^{-E}$, and $x_{c}^{b}=e^{-2E}$. The solution of Equation (9) is drawn by the solid line in Figure 10a, together with the $\phi_{B}$ calculated directly from the simulations. A reasonable consistency between the simulation and theoretical data was shown to be evident.

The particle-based description can also provide an analytical expression for $\mu_{p}$, which is expressed as follows:

(10) $$\begin{array}{c} \frac{\mu_{p}}{\kappa_{B}T}\propto -m\phi_{B}\ln\left\{1+{\phi_{B}}^{m/2}{\left(\frac{x_{c}}{x_{c}^{b}}\right)}^{1/2}+\left(\frac{x_{c}}{x_{c}^{a}}\right)\right\}. \end{array}$$

A good agreement is obtained by simply inserting the values for *m* in Equation (10); see Figure 10b.

We wish to highlight that a more generalized picture of cosolvent cluster binding was also formulated in [89]. Moreover, we only discuss the simplest picture that can capture the leading-order contribution to the generic microscopic picture of co-nonsolvency in our review. Note as well that, even when the theoretical framework based on the Langmuir-like adsorption isotherm was initially proposed for polymer solutions [88,89], it was later extended to study the co-nonsolvency behavior of polymer brushes [69,94,95].

## 3. Cosolvency

**Figure 11:**
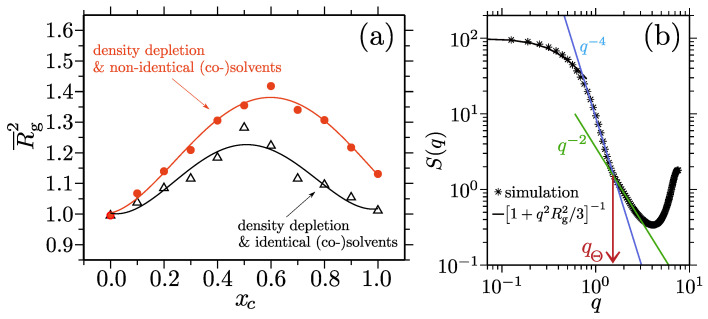
Part (**a**) shows the normalized squared radius of gyration ${\bar{R}}_{g}^{2}=\langle R_{g}^{2}\rangle /\langle R_{g}^{2}\left(x_{c}=0\right)\rangle$ as a function of cosolvent mole fraction $x_{c}$. Results are shown for the generic simulations and for two different cases described in the legends. $\langle R_{g}{\left(x_{c}=0\right)}^{2}\rangle =2.6\pm 0.4\sigma^{2}$ and ${\bar{R}}_{\Theta }^{2}=2.13$ with ${\bar{R}}_{\Theta }=R_{\Theta }/R_{g}\left(x_{c}=0\right)$ define the normalized Θ-point gyration radius. The orange data set closely mimics PMMA behavior in aqueous alcohol mixtures. Part (**b**) illustrates single-chain form factor S(q), which shows the conformations for $x_{c}=0.0$ and $x_{c}=0.7$ in the orange dataset of part (**a**). The black line represents the Guiner region for q→0, and the vertical arrow indicates the effective Θ-like blob size at $q=q_{\Theta }$. Datasets have been taken from Ref. [131].

In contrast to co-nonsolvency, one also observes the phenomenon that a polymer swells in the mixtures of two poor solvents. This phenomenon is commonly referred to as cosolvency [125], which is typically observed for PMMAs in mixtures of water and short alcohols (such as methanol, ethanol, propanol, and/or isopropanol) [126,127,128]. PMMAs also show cosolvency in 2-butanol and 1-chlorobutane mixtures [125]. Additionally, poly(N-(6-acetamidopyridin-2-yl)acrylamide) (PNAPAAm) [129] and corn starch [130] also show cosolvency in aqueous alcohol mixtures. For example, when both water and alcohols are poor solvents for PMMAs, the mixture is a somewhat better solvent that attains a maximum around $x_{c}≃0.7$; see the orange dataset in Figure 11a and the experimental data in Refs. [126,127,128].

In a standard poor solvent (i.e., in contrast to the co-nonsolvency-based collapse described above) the effective attraction between the two monomers of a polymer can be seen as a depletion-induced attraction, which is a phenomenon that is well-known in colloidal science [132,133,134,135,136]. More specifically, monomer–monomer attraction will occur in a system of purely repulsive components if the monomer–solvent repulsion (or excluded volume) becomes larger than the monomer–monomer repulsive interaction. In this case, the resultant single-chain structure in a dilute solution can be well-described by the sphere scattering following $S\left(q\right)\propto q^{-4}$ [12,13,14]. This picture holds for PMMA behavior in pure water or in pure alcohol. However, when a polymer swells within the intermediate solvent–cosolvent mixing ratios, this does not mean that a polymer opens up to a fully expanded coil. Instead, the generic simulation data on cosolvency proposes that, while a chain remains globally collapsed around $x_{c}≃0.7$ (shown by $S\left(q\right)\propto q^{-4}$ in Figure 11b), it consists of Θ blobs with typical sizes of $\ell_{\Theta }≃4.5\sigma$ for this specific set of parameters (shown by $S\left(q\right)\propto q^{-2}$ in Figure 11b) [131].

What causes the swelling of a polymer in mixed poor solvents? This was answered within the framework of depletion-induced attraction [131]. For example, the magnitude of depletion interaction has been directly related to the total ρ of the bulk solution [132,133,134,135,136]. Therefore, when a polymer swells, there should be some indication via the ρ around $x_{c}≃0.7$. Indeed, a closer look at the aqueous alcohol mixtures reveals that the ρ shows a dip in ρ [131,137] to attain a minimum around $x_{c}≃0.5$. The larger the alcohol is, the larger the deviation that is measured from the linear interpolation of the ρ. This deviation is a key factor with respect to swelling a polymer, because the reduction in the ρ also reduces the repulsive forces between a polymer and the bulk solution components. Furthermore, while the identical polymer–solvent and polymer–cosolvent interactions swell a polymer at $x_{c}≃0.5$ (see the black dataset in Figure 11a), the nonidentical interactions lead the solvation peak to shift towards a higher $x_{c}$ (see the orange dataset in Figure 11a) [131]. In summary, while the pure poor solvent-driven collapse can be viewed as a depletion-induced attraction, the swelling is due to the “depleted depletion” because of the bulk solution behavior.

### Flory–Huggins Mean Field Theory

The cosolvency phenomenon is not only an opposite effect to that of the co-nonsolvency phenomenon; it can also be explained within the Flory–Huggins-type mean field description. As discussed in the preceding paragraph, cosolvency naturally emerges at a constant pressure *P* and is dictated by the dip in the ρ. For this analytical treatment, we consider $\phi_{p}\to 0$, and, thuss the majority of the system volume is occupied by solvent–cosolvent mixtures. Therefore, it can be treated within a simplified limit of the binary mixture. In this case, the total free energy can be written as follows [131]:

(11) $$\begin{array}{ccc} \frac{F}{\kappa_{B}T} & = & \frac{F_{s}\left(v\right)}{\kappa_{B}T}\\ & + & x_{c}\ln\left(x_{c}\right)+\left(1-x_{c}\right)\ln\left(1-x_{c}\right)\\ & + & \chi_{sc}\left(v\right)x_{c}\left(1-x_{c}\right), \end{array}$$

where $F_{s}\left(v\right)$ and $\chi_{sc}\left(v\right)$ are the molar volume (*v*) dependent-free energy of the pure (co)solvent and solvent–cosolvent interaction parameters, respectively. For a given *P*, *v* is thus controlled by the following:

(12) $$\begin{array}{c} P=P_{s}\left(v\right)-\kappa_{B}Tx_{c}\left(1-x_{c}\right)\frac{\partial \chi_{sc}\left(v\right)}{\partial v}, \end{array}$$

with $P_{s}\left(v\right)=-\partial F_{s}/\partial v$ being the pressure of the reference system. For a small variation in *v*, one obtains $v=v_{o}\left[1+\zeta \;x_{c}\left(1-x_{c}\right)\right]$, where

(13) $$\begin{array}{c} \zeta =\frac{\kappa_{B}T}{v}\frac{\partial \chi_{sc}\left(v\right)}{\partial v}{\left[\frac{\partial P_{s}\left(v\right)}{\partial v}\right]}^{-1}, \end{array}$$

which measures the relative sensitivity of the interaction parameter and reference pressure to *v*. The change in $\chi_{sc}$ between the constant density and the constant pressure ensembles can then be estimated using the following:

(14) $$\begin{array}{ccc} \chi_{sc}\left(v\right) & = & \chi_{sc}\left(v_{o}\right)\\ & + & v\frac{\partial \chi_{sc}\left(v\right)}{\partial v}{|}_{x_{c}\to 0}\zeta x_{c}\left(1-x_{c}\right). \end{array}$$

Since $v\partial \chi_{sc}\left(v\right)/\partial v∼\zeta$, the $\chi_{sc}$ obtained between different ensembles is only perturbed to the second order in ζ. Here, for $x_{c}=0.5$, the $\chi_{sc}$ only changes by ∼11% between two ensembles [131], which might look a bit small. However, considering that a polymer does not swell in the mixed poor solvents, an estimate of 11% is reasonable using Equation (14).

## 4. Design of Complex Copolymers in Mixed Solvents

The constant quest towards the design of polymer (or “smart” polymer) architectures for a range of advanced functional materials is at the heart of establishing a functional understating of their structure–property relationship [138,139,140,141]. In this context, the solution processing of these systems has a wide range of practical applications in biomedical encapsulation [38,39], artificial muscle tissues [19,139,142], “pick-up and place” systems [143], and biomedical glues [141]. These applications usually require a careful variation of the external stimuli. For this purpose, a variety of copolymer architectures are proposed here that can be used for a better conformational predictability and, thus, a better control on their functionalities. This includes sequences of smart polymers [16,18,36,84,144], copolymer sequences of smart and standard polymers [22,43], conventional copolymers [37], pluronics [37,39], and/or elastin-based peptides [15,77,78].

**Figure 12:**
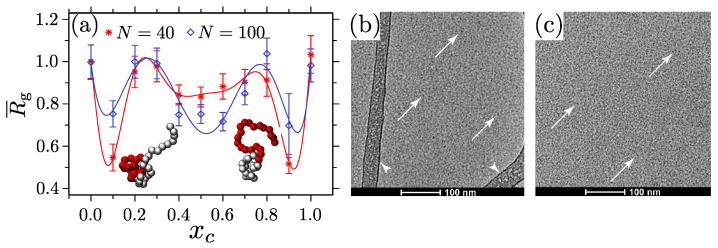
Part (**a**) shows the normalized radius of gyration ${\bar{R}}_{g}\left(x_{c}\right)/{\bar{R}}_{g}\left(x_{c}=0\right)$ for a diblock copolymer as a function of cosolvent mole fraction $x_{c}$ for two different chain lengths $N_{\ell }$. Data are shown for the generic model for infinite polymer dilution, i.e., a single-chain conformation. The generic model mimics the conformations of a diblock consisting of poly(N-isopropylacrylamide) (PNIPAM) and a poly(2-(methacryloyloxy)ethylphosphorylcholine) (PMPC) blocks in aqueous alcohol mixtures. The simulation snapshots are shown for $x_{c}$ = 0.1 (when the PNIPAM block collapses, shown as red beads) and for $x_{c}$ = 0.9 (when the PMPC block collapses, shown as silver beads). Parts (**b**,**c**) show the Cryo–TEM images of p(NIPAm–co–MPC) for two ethanol volume fractions: (**b**) 0.04 and (**c**) 0.74, respectively. The core of the polymer micelles are highlighted by the white arrows. These figures have been reproduced with permission from the American Chemical Society [144].

The temperature responsiveness is a commonly used external stimulus for tuning conformations in aqueous environments [16,18,22,37,39,40,145,146,147,148]. However, a lesser-investigated topic is a design principle using cosolvent effects [77,78,84,144,149]. This is particularly the case because a grand challenge here is to find a multicomponent copolymer sequence where different monomer units show contrasting conformational behavior in the same solvent–cosolvent mixtures. In this context, one possible combination may be the copolymers consisting of PNIPAM and poly(2-(methacryloyloxy)ethylphosphorylcholine) (PMPC) in aqueous alcohol mixtures [144]. This system is interesting because, while PNIPAM shows co-nonsolvency in the water-rich region [53,54], PMPC collapses in the alcohol-rich region [57]. This leads to an interesting conformational behavior of a single chain that results in p(NIPAm–co–MPC); see the simulation data in Figure 12a. The direct consequence of this behavior is the observation of reversible miscellization at a finite concentration of the p(NIPAm–co–MPC); the Cryo–TEM measurements in Figure 12b,c show the micelle formation.

Random sequences of the PNIPAM and PMPC show even more interesting conformations depending on the (co)solvent interaction contrast, the sequence lengths, and the monomer types; see the Supplementary Figure in Ref. [144]. Another possible system is a combination of monomers where one block shows co-nonsolvency, and the other shows cosolvency. One such example is a PMMA–PNIPAM diblock copolymer in aqueous alcohol mixtures [149]. In this case, the PNIPAM block collapses in the water-rich region (i.e., co-nonsolvency occurs) [53,54], and the PMMA block swells in the alcohol-rich region (i.e., cosolvency occurs) [126,127,128].

## 5. Heat Flow in Smart Polymers

In the preceding sections, we have presented an overview of the solution processable “smart” responsive polymers. Here, the applications of smart polymers are not only restricted to their dilute solutions; rather, they are also used for the thermal switching and also in designing lightweight high-performance organic solids. The main goal here is to provide a guiding path toward designing a set of biocompatible commodity polymeric materials with tunable thermal properties [150,151,152,153,154]. In this context, one of the central properties that often dictates the broad applicability of smart polymer or polymer-based materials is the ability to conduct the heat current, as quantified by the thermal transport coefficient κ [150,154]. For example, when a material is used under high-temperature conditions, such as the organic solar cells, the electronic packaging, and/or the heat sinking systems, a high κ is needed [151,152,155]. Low κ materials are required for their possible use in thermoelectrics [156]. Therefore, this poses a need to achieve a predictive tunability in κ.

Traditionally, most studies on thermal transport have attempted to unveil the structure–property relationship in nanomaterials [157,158,159,160,161,162]. Recent interest has been devoted to studying κ behavior in macromolecular systems [151,152,153,155,163,164,165,166,167,168,169] because of the inherent advantage of polymers in designing advanced functional materials. Therefore, in the following, we will highlight two related, yet distinct, topics that are relevant in the context of the heat flow in organic materials: (1) thermal switching controlled via coil-to-globule transition and (2) the heat flow in organic solids.

### 5.1. Thermal Switching in Smart Responsive Polymers

Heat management in advanced materials for a broad range of applications requires a very delicate control of their thermal switching. These applications span across thermoelectric energy conversion, energy storage, space technology, and sensing. Traditionally, the performance of thermal switches often suffers from slow transition rates [165,166,170]. Here, thermoresponsive smart polymers may serve as the suitable candidates. Particularly, a slight change in the external stimulus can significantly alter the structure, function, and stability of the smart polymers and, thus, may be used for fast switching in these systems.

**Figure 13:**
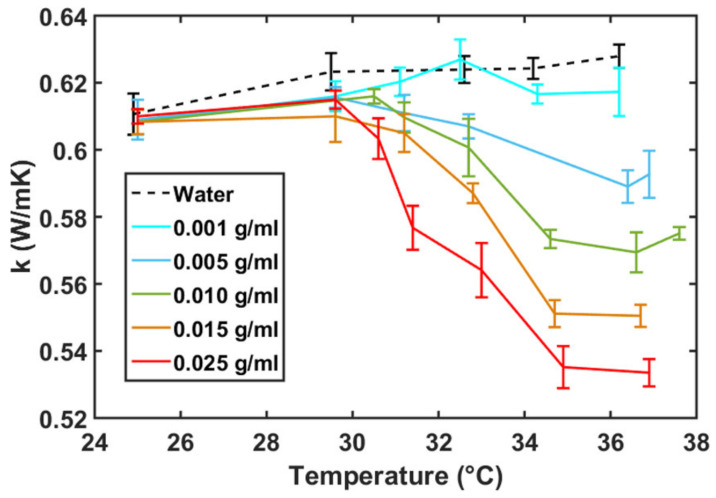
The thermal transport coefficient κ as a function of temperature *T* for the aqueous poly(N–isopropylacrylamide) (PNIPAM) solutions with changing PNIPAM concentrations. This figure has been reproduced with permission from the American Chemical Society [165].

In recent times, the studies of the κ in polymer solutions have gained attention [165,166,171]. In particular, the conformational transition of PNIPAM [165] and PNIPAM-based hydrogels [166] in water have been shown to play a key role in dictating the heat flow. For example, Figure 13 shows that the κ of aqueous PNIPAM solutions follows the same trend as their LCST coil-to-globule transition, with $T_{\ell }≃305$ K (or 32 °C) [5,21]. On the contrary, however, an opposite trend (i.e., the κ increases above $T_{\mathrm{cloud}}$) has been observed for concentrated polymer solutions [172]. These distinct results show that the polymer concentration plays a key role in dictating the κ behavior in polymer solutions.

Typically, κ increases with increasing *T* in disordered systems, which is because of increased localized vibrations [173,174]. This behavior has been weakly observed for pure water data; see the black dataset in Figure 13. The rather counterintuitive κ behavior, i.e., the κ decreases with increasing *T*, can possibly be explained by the loss of hydrogen bonds and the resultant breaking of the water caging around a polymer [165,166]. This further consolidates the fact that there is a direct correlation between the conformation and the κ value. Here, however, it should be noted that the change in κ with *T* in PNIPAM solutions may not be solely due to the conformations; rather, a delicate balance between the conformation, water tetrahedrality near a polymer, and hydrogen bonding leads to the data obtained in Figure 13, which, to the best of our knowledge, is a rather open discussion.

### 5.2. Smart Polymers for Organic Solids

Organic solids (i.e., amorphous polymeric solids) are another class of materials where the H-bonded nature of the smart polymers can be used to tune their κ behavior. However, organic solids fall under the category of “low-κ” materials [150,151,152,154]. For example, when polymer properties are dictated by weak vdW interactions κ≤0.2 W/Km, as is the case for PMMA, PS, and PAP [150,152,154,175]. In H-bonded systems (or “smart polymers”) where κ→0.4 W/Km, examples include PAA, PAM, PNIPAM, and PVA [151,152,176]. If we put the above κ values in perspective, a single-carbon nanotube (CNT) has $\kappa \geq 10^{3}$ W/Km [177], i.e., it is four orders of magnitude larger than the standard amorphous polymers. Attempts have been made to increase the κ of polymers by blending in high-κ materials, such as CNT or fullerene, within the amorphous polymers. This, however, requires the concentrations of the high-κ component to be larger than their percolation thresholds, and, thus, the original polymeric system looses all its flexibility. Therefore, a better alternative approach is to use the microscopic polymer interactions, conformation, morphology, and bond properties to achieve a tunable κ in polymers.

**Figure 14:**
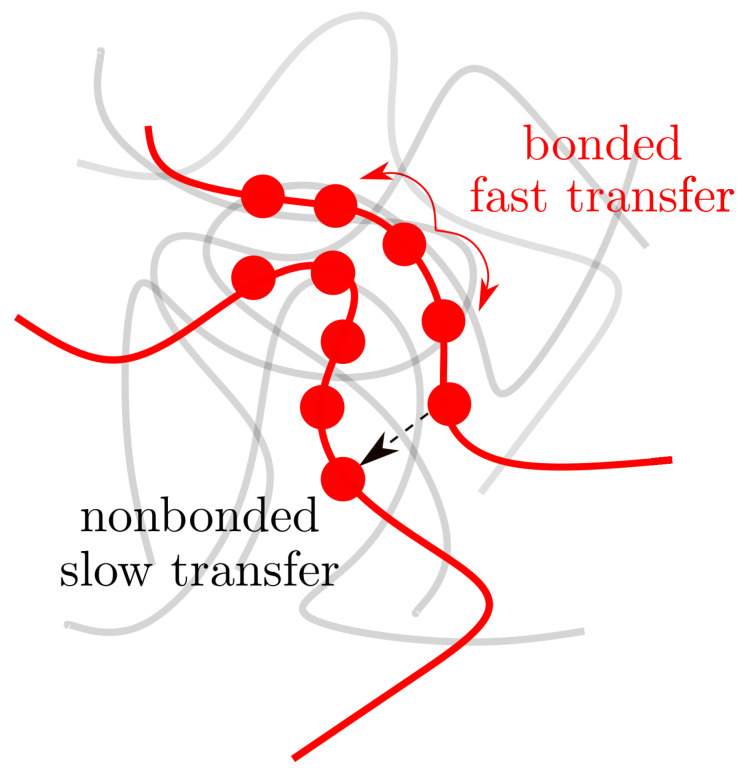
A schematic representation of the energy transfer pathways between two bonded (red arrows) and between two nonbonded (black arrow) monomers.

Polymers are a special case, because even when the macroscopic κ remains rather small, they have different microscopic pathways of energy transport [178,179]. More specifically, in an organic solid consisting of linear polymers, the energy can be transferred between two nonbonded monomers and between two covalently bonded monomers. A simple schematic of this energy transfer scheme is presented in Figure 14. Here, it is known that the rate of energy transfer between two bonded monomers is about 50–100 times faster than between two nonbonded monomers [163,179,180]. This is because the stiffness of a covalent contact is larger than 250 GPa [181], while the stiffness of a system that is purely dominated by nonbonded interactions may range between 2–5 GPa (vdW or H–bond) [152]. In this context, it is known that the stiffness is directly related to the κ [157,162] and, thus, is consistent with the faster energy transfer between the bonded monomers [179], as is evident from chain-oriented systems [163,180].

A simple model that connects the κ with the material stiffness is the minimum thermal conductivity model [157]:

(15) $$\kappa^{\min}={\left(\frac{\pi }{432}\right)}^{1/3}k_{B}^{1/3}c^{2/3}\left(v_{\ell }+2v_{t}\right),$$

where *c* is the heat capacity. $v_{l}=\sqrt{C_{11}/\rho_{m}}$ and $v_{t}=\sqrt{C_{44}/\rho_{m}}$ are the longitudinal and transverse sound wave velocities, respectively. $C_{11}=K+4C_{44}/3$, $C_{44}$ is the shear modulus, *K* is the bulk modulus, and $\rho_{m}$ is the mass density.

Equation (15) clearly indicates that, if the sound wave propagation (or stiffness) in a sample is tuned, it also helps with tuning the κ. Recently, tuning the κ values of organic solids has attracted great attention. This has been achieved via macromolecular engineering, which has included the following: (1) tuning the microscopic (nonbonded) interactions in linear polymers and polymer blends [150,151,152,153,154,182] and (2) by using covalent bonds [163,167,168,169,175,180,183,184].

#### 5.2.1. Tuning κ via Nonbonded Interactions

**Figure 15:**
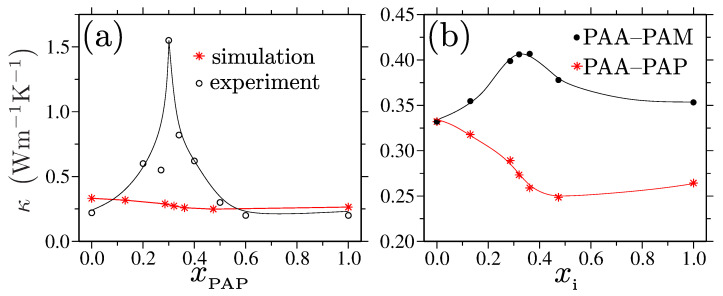
Part (**a**) shows the thermal transport coefficient κ for the blends of poly(acrylic acid) (PAA) and poly(N-acryloyl piperidine) (PAP) as a function of the PAP monomer mole fraction $x_{{}_{\mathrm{PAP}}}$ [151]. Note that a separate set of experiments did not observe this large enhancement in κ in PAA–PAP blends [152]. Part (**b**) shows a comparative data for PAA–PAP and PAA and polyacrylamide (PAM) blends. These figures have been reproduced with permission from the American Chemical Society [153].

A common protocol to tune the κ is by tuning the nonbonded interactions. Therefore, this poses a need to look beyond the standard single component polymeric systems, where the κ values remain rather restricted because of their predefined microscopic interactions. Here, a polymer blend with the desired combinations of polymers serves as a better candidate. In particular, a recent experimental study measured the κ→1.5 W/Km in an asymmetric blend consisting of short PAP and long PAA chains [151]; see the experimental data in Figure 15a. This κ enhancement was also shown to be directly related to an enhancement in the glass transition temperature $T_{g}$ for a blend in comparison to the $T_{g}$ of both of the individual components.

The main advantage of using such asymmetric systems is that they form a H-bonded crosslinked network, where the PAP molecules crosslink two PAA monomers from two different chains. A prerequisite of this protocol is that the PAP and PAA remain perfectly miscible over all monomer molar concentrations $x_{{}_{\mathrm{PAP}}}$. While the experimental data in Ref. [151] are extremely promising and show a significant improvement in the κ values, another independent set of experiments has argued that the PAP and PAA phases separate within a range of $x_{{}_{\mathrm{PAP}}}$, where a large enhancement in the κ was observed and where the κ<0.4 W/Km over the full range of $x_{{}_{\mathrm{PAP}}}$ [152]. The later experimental finding was also confirmed by the simulation data where no enhancement in the κ was observed [153]; see the simulation data in Figure 15a.

Going beyond these experiments, complementary simulation results also showed that the phase separation in PAP–PAA blends is triggered by the stacking of the aromatic side groups of different PAP molecules. Therefore, by introducing a modification in the monomer structure, i.e., by replacing the PAP with PAM, the miscibility of PAM–PAA blends can be greatly improved [153]. Even in this case, the maximum attainable κ remained below 0.4 W/Km; see the black dataset in Figure 15b. These results further consolidate the fact that there might be an absolute maxima in the attainable κ of the amorphous organic solids consisting of neutral linear polymers or polymer blends, which is directly related to the limitations of the maximum attainable $v_{\ell }$ and $v_{t}$ values [152]. This observation is independent of the specific chemical details and of the $T_{g}$, thus showing that neither of these system-specific quantities plays a key role in controlling the κ [176].

Typical systems where the κ may be improved beyond the maximum limit of 0.4 W/Km include, but are not limited to, polyelectrolytes, semicrystalline polymers, and/or polymers under high pressures. In the case of polyelectrolytes, it was shown that the change in the degree of ionization could increase the κ→0.6 W/Km for PAA [185], which was attributed to improved vdW forces due to electrostatic attractions. A similar argument may also hold for the case when a polymeric system is subjected to high pressures [186], where κ→1.0 W/Km. Other examples include the systems consisting of complex monomer structures, where π−π stacking leads to semicrystalline orders and, thus, to potentially increasing the κ [155,156]. Within the same spirit, polypeptides can also be used, where the fraction of the secondary structure (or the degree of crystallinity) leads to a significant increase in the κ [164].

#### 5.2.2. Tuning κ via Bonded Interactions

Taking motivation from the observed significantly higher energy transfer rates between bonded monomers in comparison to nonbonded monomers, systems have been synthesized where the properties were dominated by bonded interactions. The systems where properties are dominated by the covalent bonds are chain-oriented systems (such as polymer fibers and molecular forests) [163,175,180] and crosslinked networks (such as epoxies and vitrimers) [167,168,169,183,184]. While the chain-oriented systems showed the κ→100 W/Km [163], the maximum attainable κ≃0.3 W/Km was observed for the epoxies [167,184]. Even when the epoxies usually have a very high density of bonds, they still have a rather small κ. This is particularly because the heat flow in epoxies is dictated by a delicate balance between bond types [167,183,184] and complex microstructures [167]. Epoxies with a certain degree of crystalline order can show a bit of a higher κ value. For example, semicrystalline epoxies have shown the κ→0.5 W/Km [168,169], and, for the liquid crystalline epoxies, they have shown the κ→1.0 W/km [187].

Note that, even though epoxies have been widely known for several decades as lightweight high-performance materials, they have found renewed attention within the last 2–3 years within the constant quest toward increasing the κ values of organic solids. Here, we only sketch a very short highlight of the heat flow in epoxies. Given that these are rather complex systems, a solid discussion is a topic of its own. This will be presented in a different note.

### 5.3. Classical Simulations and Comparing κ with the Experimental Data

Lastly, we would like to briefly comment on the calculated $\kappa_{\mathrm{cla}}$ within classical setups and their comparisons with the experimental data $\kappa_{\exp}$. In this context, the results indicate that the $\kappa_{\mathrm{cla}}>\kappa_{\exp}$ [176,188,189]. This is the case even when the simulations have been conducted using the most accurate (available) empirical potentials, as well as when the $\kappa_{\mathrm{cla}}$ values have been calculated very carefully. Here, it is important to highlight that this discrepancy between the $\kappa_{\mathrm{cla}}$ and $\kappa_{\exp}$ is not a technical issue; rather, it is a more fundamental issue. For example, as highlighted in Equation (15), the heat flow is directly related to the *c* and *v*. If any of the *c* and *v* are calculated inaccurately, this automatically leads to the wrong $\kappa_{\mathrm{cla}}$ estimates. In this context, one of the major contributions to the error in $\kappa_{\mathrm{cla}}$ calculations is the inaccurate estimates of the *c* within classical simulation setups [190,191,192].

**Figure 16:**
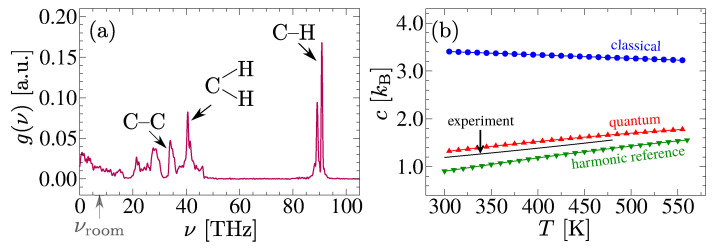
Part (**a**) shows the vibrational density of states g(ν) for hexadecane calculated using Equation (17). A few vibrational modes are highlighted as the legends. Part (**b**) shows a comparison between the specific heat *c* calculated in simulations by different methods and measured in experiments. These figures have been reproduced with permission from the American Physical Society [190].

Within the classical description, the Dulong–Petit limit yields $c=3Nk_{B}$, i.e., all modes contribute equally to the total *c*. In reality, however, many modes in polymers are quantum-mechanically frozen at the room temperature of $T_{\mathrm{room}}=300$ K. For example, the C–H vibrational frequency (a common building block of polymers) is ν≃90 THz, while the $\nu_{\mathrm{room}}≃6.2$ THz at $T_{\mathrm{room}}$ [190]; see the vibrational density of the states g(ν) for an alkane system in Figure 16a. Similarly, there are other modes, such as the C–C bond and the H–C–H angular vibrations, that have ν values that are significantly larger than a $\nu_{\mathrm{room}}≃6.2$ THz. Even when these modes are excited at $T_{\mathrm{room}}$, their contributions are automatically incorporated into the *c* and, thus, affect the $\kappa_{\mathrm{cla}}$ estimates. Therefore, unless the $\kappa_{\mathrm{cla}}$ is reweighted to account for the quantum corrections to *c*, reasonable comparisons may be difficult to achieve within the classical setups.

An earlier experimental study proposed a simplified correction to *c* in Equation (15). This approach proposed a corrected estimate using $c_{\mathrm{cor}}=3N_{\mathrm{cor}}k_{B}$, where $N_{\mathrm{cor}}=2\left(N-N_{H}\right)/3$ accounts for the contributions of the stiff modes [186], and $N_{H}$ is the number of hydrogen atoms in a system. While this approximation gives reasonably accurate estimates [157,186], it will also be important to have a method that uses a solid theoretical basis.

One method to calculate the $c_{\mathrm{cor}}$ is the harmonic reference proposed in Ref. [193]. Here, if the frequency of a (stiff) harmonic mode is known, this mode contributes $k_{B}{\left(h\nu /2k_{B}T\right)}^{2}$/$\sinh^{2}\left(h\nu /2k_{B}T\right)$ to the $c_{\mathrm{cor}}$. Then, the total equates to the following:

(16) $$c_{\mathrm{cor}}\left(T\right)=k_{B}\int_{0}^{\infty }\frac{{\left(h\nu /2k_{B}T\right)}^{2}}{\sinh^{2}\left(h\nu /2k_{B}T\right)}g\left(\nu \right)d\nu .$$

Within the harmonic approximation, the g(ν) can be calculated using the following formula:

(17) $$g\left(\nu \right)=\frac{1}{G}\int_{0}^{\infty }\;\;dt\;\cos\left(2\pi \nu \Delta t\right)\;\frac{C(\Delta t)}{C(0)},$$

where $C\left(\Delta t\right)=\sum_{n}m_{n}\;\langle v_{n}\left(t\right)\cdot v_{n}\left(t+\Delta t\right)\rangle$ is the mass-weighted velocity autocorrelation function, and *G* ensures that $\int_{0}^{\infty }g\left(\nu \right)d\nu =1$. Note that, in a harmonic system, the C(Δt) results from the superposition of individual normal modes and their Fourier transforms make it possible to determine what percentage of modes have what resonance frequencies. The result using Equation (16) showed a good agreement with the experimental data; see the green data set (harmonic reference) in Figure 16b.

A more recent method, particularly tuned for the liquid polymers, used Equation (16) to estimate the difference between the classical and the quantum systems by using the following equation:

(18) $$\Delta c_{\mathrm{rel}}\left(T\right)=\int_{0}^{\infty }\left\{1-\frac{{\left(h\nu /2k_{B}T\right)}^{2}}{\sinh^{2}\left(h\nu /2k_{B}T\right)}\right\}g\left(\nu \right)d\nu ,$$

and the method then used $c_{\mathrm{cor}}\left(T\right)=c_{\mathrm{cla}}\left(T\right)-\Delta c_{\mathrm{rel}}\left(T\right)$ [190]. Here, $c_{\mathrm{cla}}=\left[H\left(T-\Delta T\right)-H\left(T-\Delta T\right)\right]/2\Delta T$, and H(T) is the enthalpy. The main advantage of the later approach is that the stiff harmonic modes are corrected, while the contributions from the anharmonic modes remain unaltered, which is absolutely important for polymeric systems. This approach also yielded a good agreement with the experimental data; see the red dataset (quantum) in Figure 16b.

The method proposed in Ref. [190] also highlighted different strategies to estimate the $c_{\mathrm{cor}}$ to account for the missing degrees of freedom (DOF) within the united-atom and/or coarse-grained models [190]. A direct implication is that a certain percentage error in the κ calculations using the united-atom models comes from the missing DOFs [173].

## 6. Concluding Remarks

The field of polymer research has grown quite significantly over the last few decades, where many new exciting topics and systems are being constantly investigated using experimental, computational, and analytical tools. Smart responsive polymers are one such exciting class that has gained tremendous attention over the last decade. These systems are not only important because of their biocompatible and thermoresponsive nature, but also because they often show counterintuitive solvation behaviors in mixtures of solvents. Therefore, it is often challenging to develop the microscopic structure–function relationship in these systems, while they have tremendous technological relevance that spans across biomedical applications [19,38,39,139,142], adhesive polymer-coated surfaces [141,143,194], and biocompatible commodity organic solids [150,151,152,153,154,156].

In this review, we have presented a brief overview of the present state of smart polymer research. We began with discussing the polymer conformations in single and multicomponent mixtures. In particular, we discussed two opposite phenomena, namely, co-nonsolvency and cosolvency. While co-nonsolvency is associated with the collapse of a polymer in the mixtures of two miscible good solvents, cosolvency is a term given to a phenomenon when a polymer swells in poor solvent mixtures. The present state of understanding proposed by the simulation and analytical tools was discussed, together with the complementary experiments. A generic picture has been presented that highlights that these two phenomena are not restricted to a few specific systems; rather, a broad range of polymers shows such solvation behavior in their respective mixtures of solvents. One system that requires special mention is the PMMA system, which shows both co-nonsolvency [79] and cosolvency [126,127,128] behaviors. We have also highlighted how smart polymeric materials may be used as the guiding path toward designing advanced materials with tunable and predictive responsiveness.

This review also highlighted the possible applications of smart polymers for biocompatible organic solids, which is a topic that has gained a lot of attention over the last decade. Within this topic, one central property of interest is the thermal conductivity coefficient κ, which is of great importance because of the possible use of organic solids for various applications. For example, amorphous organic solids consisting of linear polymers or linear polymer blends have very low κ values that may provide a good material choice when they are used as thermoelectrics. However, this low-κ behavior also poses great problems when an organic solid is used under high-temperature conditions. Therefore, a large number of works has devoted significant efforts to achieving a large κ for organic solids via macromolecular engineering. This has ranged from tuning the nonbonded interactions in polymer blends to employing highly crosslinked polymer networks where bonded interactions are dominant.

Lastly, we want to conclude by commenting that, even though we briefly covered two distinct applications of smart polymers, it was still impossible to cover all aspects of this exciting research topic. More specifically, we have predominantly based our discussions on neutral polymers in mixed solvents and in their amorphous solid states. However, there are several other topics that are important within the smart polymer research, especially when dealing with the polymers in ionic solutions, electrolytes, organic electronics, and semicrystalline and liquid crystalline epoxy networks. These are only a few (out of many) exciting systems that we could not cover in this review, but we hope that the discussion presented here might provide some basics regarding the present state of smart polymer research that may stimulate further discussions on this topic.
